# DNA methylation from birth to late adolescence and development of multiple-risk behaviours

**DOI:** 10.1016/j.jad.2017.11.055

**Published:** 2018-02

**Authors:** F. de Vocht, M. Suderman, K. Tilling, J. Heron, L.D. Howe, R. Campbell, M. Hickman, C. Relton

**Affiliations:** a School of Social and Community Medicine, University of Bristol, Bristol, UK; b MRC Integrative Epidemiology Unit, UK

**Keywords:** ALSPAC, DNA methylation, Epigenetics, Risk behaviour, Multiple risk behaviour, Adolescents

## Abstract

**Background:**

Risk behaviours in adolescence are linked to poor educational attainment and health and other outcomes in young adulthood. We explored whether there are molecular mechanisms associated with the development, or the result, of multiple risk behaviours (MRBs).

**Methods:**

MRBs (antisocial behaviour and delinquency, traffic-related risk behaviour, risky sexual behaviour, lack of exercise) and their sumscore were characterized based on self-reported questions at age 7 and 17 within the ARIES subsample of the ALSPAC birth cohort, and were linked to DNA methylation at over 485,000 CpG sites at ages 0,7 and 17. Associations were determined for participants with complete data (n = 227–575).

**Results:**

There was weak evidence of associations between cumulative MRBs and methylation at cg01783492 and cg16720578 at age 17. DNA methylation at age 17 was associated with risky sexual behaviour (cg22883332), lack of exercise (cg03152353, cg20056908, cg20571116) and substance use (cg02188400, cg13906377). No associations between DNA methylation and individual risk behaviours at age 7 were observed. DNA methylation at age 7 might predispose for traffic-related risk behaviour (cg24683561) and substance use (cg08761410) at age 17.

**Limitations:**

Main limitations are absence of information on directly measured blood cell type proportions and tissue specificity, and a modest sample size.

**Conclusions:**

Cumulative MRB in late adolescence was associated with effects on DNA methylation. More specifically, risky sexual behaviour and sedentary behaviour are associated with changes in DNA methylation, while DNA methylation in childhood may predict later traffic-related risky behaviour. For substance use effects in both temporal directions were observed.

## Highlights

• This study is the first to provide evidence that MRB behaviour affects DNA methylation.
• This study allowing for assessment of temporal associations across the early lifecourse.
• Risky sexual behaviour and sedentary behaviour may induce DNA methylation changes.
• DNA methylation in childhood may predispose for later traffic-related risky behaviour.
• Associations in both temporal directions were observed for substance abuse.

## 1 Introduction

Risk behaviours in adolescence, such as substance use, alcohol consumption, poor diet, physical inactivity, unprotected sex and antisocial behaviour are common (Kipping et al., 2015), and associated with adverse health and other outcomes in later adolescence (Hale and Viner, 2016). Analysis of a UK birth cohort (the Avon Longitudinal Study of Parents and Children cohort study) showed that multiple risk behaviours (MRBs) in adolescence are common, with 74% of 15–16 year olds being physically inactive, 34% consuming alcohol at hazardous levels, and 42% being engaged in antisocial and criminal behaviour (MacArthur et al., 2012). Initiation of most risk behaviours generally occurs at ages 14–16 (Hale and Viner, 2016), while it has further been shown that those who initiate risk behaviours early were more likely to be multiple risk-takers (DuRant et al., 1999).

These risky behaviours are linked to poor educational attainment and a range of morbidity and mortality outcomes in later life (Galambos and Tilton-Weaver, 1998, Rohde et al., 2001, Sandfort et al., 2008, Viner and Taylor, 2007), which has made the prevention of health risk behaviours by adolescents a focus for policy in the UK and internationally (Hale and Viner, 2012). It has been shown that risk behaviours, such as for example substance use, sexual risk and delinquency in adolescence (Hair et al., 2009, Hale and Viner, 2016, Jackson et al., 2012, Meader et al., 2016, Wiefferink et al., 2006) often cluster. Two broad theories have been proposed that may explain the correlations between adolescent risk behaviours: [1] ‘*Gateway theory*’ (Pudney, 2003), which suggests that engagement in one form of risk leads to others through a decrease in perceiving dangers and/or through increased exposure to other risk behaviours, and ‘*Problem Behaviour theory*’ which purports that sets of behaviours that are defined as problematic and/or unconventional are enacted as a manifestation of disregard of social conventions or, depending on age, of maturity (Jessor et al., 1998). The implications of either theory throughout adolescence are that whereas Gateway theory predicts accumulating associations between risk behaviours with age, Problem Behaviour theory may result in a stable risk behaviour in late adolescence. This pattern has, for example, been observed for trajectories of substance abuse from early adolescence to adulthood which described how correlations between risk behaviours decrease with age, and similar patterns have been observed for drug use and sexual behaviour, but not for alcohol use (Hale and Viner, 2016). This decrease can be ascribed to a transition from general risk behaviour aetiology in adolescence to risk-specific influences in adulthood (Vrieze et al., 2012). Like most complex phenotypes, in addition to shared social/environmental factors, there may also be also be shared biological factors that influence the development of these multiple risk behaviours, and there is evidence that the generalized risk may be directly influenced by modifiable molecular factors in childhood or adolescence (Vrieze et al., 2012). In support of this, epigenetic processes have been shown to be associated with substance use in adolescents (Cecil et al., 2016).

Epigenetics, the study of heritable changes in gene expression not due to changes in DNA sequence, offers the potential to identify molecular mechanisms by which environmental and lifestyle exposures may affect health (Florath et al., 2014, Hannum et al., 2013). Epigenetic mechanisms include DNA methylation, histone modifications and microRNA, all of which act in concert to regulate gene expression (Groom et al., 2011). DNA methylation, the addition of methyl groups to nucleotide bases, is the most stable and most readily quantifiable epigenetic marker and has thus become the most widely studied. DNA methylation is affected by genetic variation (Gaunt et al., 2016), is sensitive to pre- and postnatal exogenous influences (de Vocht et al., 2015), and has also been linked to physical and psychiatric disorders, including addiction (Cecil et al., 2015). Recent technological advances have allowed the application of genomic technologies to epigenetics, facilitating the large scale generation of quantitative DNA methylation data across the genome (Mensaert et al., 2014).

In this study, we explored, for the first time, associations between multiple risk behaviours and DNA methylation measured at multiple time points across childhood, at birth, age 7 and age 17. Critically, two of the DNA methylation measurements were taken prior to the behaviour which enables investigation of the temporal direction of the associations to address the question; are epigenetic changes observed at different time points risk factors for behaviour or are they consequences of the behaviour itself?

## 2 Methods

This study used DNA methylation data generated under the auspices of the Avon Longitudinal Study of Parents and Children (ALSPAC) (Boyd et al., 2013, Fraser et al., 2013). ALSPAC recruited 14,541 pregnant women with expected delivery dates between April 1991 and December 1992. Of these initial pregnancies, there were 14,062 live births and 13,988 children who were alive at 1 year of age. The study website contains details of all the data that are available through a fully searchable data dictionary (http://www.bris.ac.uk/alspac/researchers/data-access/data-dictionary). As part of the ARIES (Relton et al., 2015) project (http://www.ariesepigenomics.org.uk), a sub-sample of 1,018 ALSPAC mother–child pairs had DNA methylation measured using the Infinium HumanMethylation450 BeadChip (Illumina, Inc.) (Dedeurwaerder et al., 2011). Here, we use DNA methylation data generated from cord blood and venous blood samples at age 7 and again at age 15 or 17 years, leading to three measurements of DNA methylation per child. All DNA methylation wet-lab and preprocessing analyses were performed at the University of Bristol as part of the ARIES project and has been described in detail previously (Relton et al., 2015).

Informed consent was obtained from all ALSPAC participants and ethical approval was obtained from the ALSPAC Law and Ethics Committee as well as Local Research Committees.

### 2.1 Outcome

Multiple risk behaviour (MRB) was characterized based on self-reported questions broadly divided into five domains: antisocial behaviour and delinquency (2 questions), traffic-related risk behaviour (4 questions), risky sexual behaviour (2 questions), sedentary behaviour (1 question), and substance use (4 questions). Where questions were not binary already, behaviours were recoded to present (1) or absent (0) based on cut-off points informed by the literature (described in Kipping et al. (2015)). Dichotomised scores for each behaviour were summed per domain to obtain a summary domain score for ages ~ 7 and ~ 17, and for age 17 these in turn were added to obtain the MRB sum score; i.e. the primary outcome of this study (range 0–13 in theory, 0–9 in this population). An overview of the questions for time point 2 (age ~ 17 years), the domains, and the different MRB scores is provided in Table 1.

**Table 1:** (Multiple) Risk Behaviour (MRB) categories at time point 2 (age ~ 17 years).

**Sum scores**	**Description**
MRB sum score (continuous)	A+B+C+D+E
MRB sum score (binary)	Low (≤ median) vs high (> median) MRB risk score
MRB sum score (25th vs 75th)	Low (≤ 25th percentile) vs high (≥ 75th percentile) MRB risk score (25th–75th percentile removed from analysis)
**Strata/individual risk behaviours**	
A) Antisocial behaviour and delinquency	1) At least once in the past year: theft from vehicle, carrying a weapon, being rude/rowdy in public place, breaking into a vehicle, taking a car that is not their own, stealing from a shop, buying stolen goods, stealing money from someone else, burgling a house, kicked/hurt someone on purpose, used a cheque book/card not their own, damaged property not their own on purpose.
	2) Has hurt themselves on purpose at least once in the last year
	3) One or more forms of gambling over past 12 months
B) Traffic-related risk behaviour	1) Has been car passenger at least once in their lifetime where the driver (a) has had alcohol or (b) does not have valid licence or (c) chose not to wear a seat belt last time travelled in a car, van or taxi.
	2) Has driven a scooter off road or w/o licence (or both) at least one time in lifetime
	3) Has driven on / off road w/o licence at least once in lifetime
	4) Did not use a helmet last time they rode a bike (within the recent four week period)
C) Risky sexual behaviour	1) Has had sexual intercourse and not used a condom on last occasion they had sex in the last 12 months
	2) Multiple sexual partners (3+) in past year
D) Lack of physical exercise	1) Has typically over the past year exercised < 5 times/wk
E) Substance use	1) Has used cannabis 2–4 times a month or more frequently in the last 12 months
	2) Typically smokes at least one cigarette per week over the last 12 months
	3) Alcohol Use Disorders Identification Test (AUDIT) score of 8 or more
	4) Illicit drug and inhalant use (excluding cannabis) over past twelve months.

### 2.2 DNA methylation profile generation

DNA was bisulphite converted using the Zymo EZ DNA Methylation™ kit (Zymo, Irvine, CA). Infinium HumanMethylation450 BeadChips (Illumina, Inc.) were used to measure genome-wide DNA methylation levels at over 485,000 CpG sites. The arrays were scanned using an Illumina iScan, with initial quality review using GenomeStudio. This assay detects methylation of cytosine at CpG islands using two site-specific probes – one to detect the methylated (M) locus and one to detect the unmethylated (U) locus. Single-base extension of the probes incorporates a labelled chain-terminating ddNTP, which is then stained with a fluorescence reagent. The ratio of fluorescent signals from the methylated site versus the unmethylated site determines the level of methylation at the locus. The level of methylation is expressed as a “Beta” value (β-value), ranging from 0 (no cytosine methylation) to 1 (complete cytosine methylation). β-values are reported as percentages.

### 2.3 Quality control

During the data generation process a wide range of batch variables were recorded in a purpose-built laboratory information management system (LIMS). The LIMS also reported quality control metrics from the standard control probes on the 450 K BeadChip. Samples failing quality (samples with > 20% probes with p-value ≥ 0.01) were repeated. Samples from all three time points in ARIES were randomized across arrays to minimise the potential for batch effects. As an additional quality control step, genotype probes on the 450 K BeadChip were compared between samples from the same individual and against SNP-chip data to identify and remove any sample mismatches.

### 2.4 Methylation profile normalisation

Raw β-values were pre-processed using R (version 3.0.1) with background correction and sub-set quantile normalisation performed using the pipeline described by Touleimat and Tost (Touleimat and Tost, 2012) and implemented in the *watermelon* R package (Pidsley et al., 2013). Finally, to reduce influence of outliers in regression models, normalized β-values were 90%-Winsorized.

### 2.5 Cell type heterogeneity

Blood is composed of many cell types and composition ratios can vary over time within a given individual as well as between individuals. DNA methylation differs between blood cell types so it is necessary to adjust for cell type variance in methylation analyses to avoid confounding. Cell type proportions for peripheral blood per individual were estimated from DNA methylation profiles using the method described by Houseman et al. (2012) using an adult peripheral blood cell type reference (Reinius et al., 2012). Cell type proportions for cord blood per individual were estimated identically but using a cord blood cell type reference (Gervin et al., 2016).

### 2.6 Statistical analyses

Associations were tested using linear models implemented by the *limma* R package (Smyth, 2004). For these analyses we only use participants with complete data. MRB sum scores were included as dependent variables as a continuous measure, as a binary score based on the median score (low (≤ 2) vs. high (> 2)), and as a comparison of extremes (25th (0–1) vs. 75th (3–9) percentiles). All models were adjusted for confounders: cell counts estimates (see above), sex, parity, birthweight, gestational age, prenatal tobacco exposure, early pregnancy alcohol exposure, maternal age, maternal BMI, maternal highest education, maternal smoking history, and paternal occupation. In addition, confounders not covered by the above were adjusted for in Surrogate Variable Analysis (discussed in detail in Leek and Storey (2007)). We conducted three cross-sectional analyses at birth, age 7 and age 17, and to explore causal directions we additionally assessed associations between MRBs at ages 0 and 7 with methylation at age 17 and conversely methylation at ages 0 and 7 with MRBs at age 17. None of the association p-values were below the 0.05 Bonferroni adjusted threshold for significance (p = 1.07 × 10^−7^). We therefore report associations at false discovery rate (FDR) less than 10% calculated using the *q* method (Storey and Tibshirani, 2003). Where associations between MRB and methylation was positive, indicating that methylation at that specific CpG site was statistically significantly more present in those individuals with MRBs compared to those without, we defined these sites as “hypermethylated” and conversely when this association was inverse, we defined these sites as “hypomethylated”.

## 3 Results

The demographics of the samples used for the different analyses are shown in Table 2. Because only participants with complete data were used, the sample sizes differed from 227 for the MRB sumscore to 575 for the analyses of ‘substance use’. The samples include slightly more women than men, and are mainly (≥ 97%) white. Participants are more often from higher socio-economic groups than the general ALSPAC population, based on information on paternal occupation and maternal and paternal education levels. About 10% of women drank alcohol during pregnancy.

**Table 2:** Demographics of ARIES participants by MRB category.

		**Sum score**	**Personal risk**	**Traffic**	**Sex**	**Exercise**	**Substance**
**N**		**227**	**441**	**319**	**524**	**364**	**575**
Sex (%)	Female	57.3	50.8	58.6	51.3	59.9	52.2
	Male	42.7	49.2	41.4	48.7	40.1	47.8
Parity (%)	0	54.6	50.6	49.5	49.0	48.4	48.0
	1	31.7	35.1	37.0	36.3	37.4	37.4
	2	10.6	10.7	10.3	11.3	10.7	11.1
	3	2.6	2.9	2.8	2.9	3.0	3.0
	4	0.4	0.7	0.3	0.6	0.5	0.5
Maternal ethnicity (%)	Non-white	2.6	2.9	3.1	2.5	3.0	2.8
	White	97.4	97.1	96.9	97.5	97.0	97.2
Paternal occupation (%)	Professional	23.8	20.0	22.3	19.3	21.4	19.1
	Managerial and technical	39.6	34.9	37.6	34.9	37.1	34.3
	Skilled manual	17.2	23.4	20.1	24.2	20.3	24.7
	Skilled non-manual	13.7	13.6	13.8	12.8	13.5	12.9
	Partly-skilled	4.4	5.7	5.3	6.3	6.0	6.8
	Unskilled occupation	1.3	2.5	0.9	2.5	1.6	2.3
Maternal education (%)	A level	33.5	29.0	32.9	29.2	31.6	29.2
	CSE	5.7	5.4	5.6	5.9	5.2	5.7
	Degree	30.4	24.7	29.5	24.2	27.7	24.5
	O level	27.3	34.5	27.9	33.8	29.9	33.6
	vocational	3.1	6.3	4.1	6.9	5.5	7.0
Paternal education (%)	A level	29.5	28.6	29.2	29.0	30.2	28.7
	CSE	8.4	11.8	9.7	11.8	10.7	12.2
	Degree	40.1	33.3	37.0	30.5	35.4	30.4
	O level	18.1	20.2	19.7	21.4	18.4	21.2
	vocational	4.0	6.1	4.4	7.3	5.2	7.5
Maternal alcohol Consumption (%) (during pregnancy)	Yes	8.8	11.3	10.0	11.5	11.3	11.7
	No	89.0	84.8	87.1	84.5	85.4	84.7

Results of the primary analyses, association between MRB sum score and DNA methylation at age 17, are shown in Table 3 and indicate weak evidence of association with hypomethylation of cg01783492, located near *TEKT5*, and hypermethylation of cg16720578, located near *BMP4*. No associations between MRB sum score and DNA methylation at age 7 with an FDR < 10% were observed.

**Table 3:** Associations between Multiple Risk Behaviour summary score at age 17 and DNA methylation at age 17.^a^

**CpG site**	**Chromosome**	**Gene**	**Genomic location**	**Position**	**Beta**^b^	**P-value**	**FDR**^c^
*Cumulative risk score*							
cg01783492	16	Downstream of *TEKT5*	–	10721112	−0.0181	3.54E−0.7	0.08
cg16720578	14	Downstream of *BMP4*	–	54410717	0.0146	1.11E−07	0.05
*Binary score (low/high)*							
None identified							
*Extreme scores (25% vs 75%)*							
None identified							

a Only CpG sites with FDR ≤ 10% reported.

b Adjusted for sex, parity, birthweight, gestational age, prenatal tobacco exposure, early pregnancy alcohol exposure, maternal age, maternal BMI, maternal highest education, maternal smoking history, paternal occupation, and surrogate variable.

c False Discovery Rate (FDR).

Stratification of the MRB score into five domains (Table 4) suggests that DNA methylation may be involved in risky sexual behaviour, lack of exercise and substance use, but is not involved in antisocial behaviour and traffic-related risk behaviour. There is weak evidence of hypomethylation of CpG site cg22883332 located on the *CALML3* gene. Sedentary behaviour at age 17 is more strongly associated with hypermethylation of three CpG sites (cg03152353, cg20056908, cg20571116) located on the *NOTCH1*, *VAMP1* and *PLCD4* genes, respectively. Substance use is associated with hypomethylation of cg02188400, located upstream of *RRAS2*, and hypermethylation of cg13906377, located upstream of microRNA 6068, respectively. Comparable analyses of DNA methylation at age 7 with accumulated risk scores for the individual domains at the same age did not result in any association.

**Table 4:** Associations between Individual Risk Behaviours and DNA methylation at different ages^a^.

**Risk behaviour**	**Age**	**CpG site**	**Chromosome**	**Gene**	**Genomic location**	**Position**	**Beta**^b^	**P-value**	**FDR**
Antisocial behaviour and delinquency	17	None							
	7	None							
	0	NA							
Traffic-related risk behaviour	17	None							
	7	None							
	0	NA							
Risky sexual behaviour	17	cg22883332	10	*CALML3*	TSS1500	5566260	−0.0335	2.18E-07	0.10
	7	NA							
	0	NA							
Lack of exercise	17	cg03152353	9	*NOTCH1*	Body	139417194	0.0245	2.80E-08	0.01
		cg20056908	2	*VAMP8*	3′UTR	85808945	0.0271	5.43E-08	0.01
		cg20571116	2	*PLCD4*	Body	219485946	0.0147	1.88E-07	0.03
	7	None							
	0	NA							
Substance use	17	cg02188400	11	Upstream of *RRAS2*	–	14406326	−0.0041	5.96E-09	0.00
		cg13906377	1	Upstream of microRNA 6068	–	63792695	0.0014	4.25E-07	0.10
	7	None							
	0	NA							

a Only CpG sites with FDR ≤ 10% reported

b Adjusted for sex, parity, birthweight, gestational age, prenatal tobacco exposure, early pregnancy alcohol exposure, maternal age, maternal BMI, maternal highest education, maternal smoking history, paternal occupation, and surrogate variable

Table 5 shows associations between DNA methylation at birth and age 7 and MRB scores, stratified by domain, at age 17 to explore whether methylation may be a risk factor for later behaviour instead of the result of behaviour. There is no evidence of association with antisocial behaviour, delinquency, risky sexual behaviour and sedentary behaviour. For traffic-related risk behaviour at age 17 in contrast, hypermethylation of cg24683561 on the *PYGO2* gene at birth and age 7 was observed, suggesting that DNA methylation predates this type of risk behaviour. Similarly, for substance use also indicate a mechanism through which cg08761410, in the first intron of *PISD*, hypermethylation may predispose for substance abuse risk.

**Table 5:** Assessing reverse causality: associations between DNA methylation at birth and age 7 and Risk behaviour at age 17.^a^

**Risk behaviour (Age 17)**	**Methylation Age**	**CpG site**	**Chromosome**	**Gene**	**Genomic location**	**Position**	**Beta**^b^	**P-value**	**FDR**
Antisocial behaviour and delinquency	7	None							
	0	None							
Traffic-related risk behaviour	7	cg24683561	1	*PYGO2*	TSS1500	154934703	0.0038	1.75E−09	0.00
	0	cg24683561	1	*PYGO2*	TSS1500	154934703	0.0037	9.49E−09	0.00
Risky sexual behaviour	7	None							
	0	None							
Lack of exercise	7	None							
	0	None							
Substance use	7	cg08761410	22	first intron *PISD*	–	32058119	0.0007	6.64E−08	0.03
	0	cg08761410	22	first intron *PISD*	–	32058119	0.0007	1.21E−07	0.06

a Only CpG sites with FDR ≤ 10% reported.

b Adjusted for sex, parity, birthweight, gestational age, prenatal tobacco exposure, early pregnancy alcohol exposure, maternal age, maternal BMI, maternal highest education, maternal smoking history, paternal occupation, and surrogate variable.

## 4 Discussion

These exploratory analyses indicate some evidence that cumulative MRB behaviour in adolescence is associated with differences in DNA methylation. In relation to specific behaviours, which we analysed to investigate whether effects in individual risk behaviours could have been concealed because our main hypothesis concerned cumulative MRB, and the direction of effects, our evidence suggests that risky sexual behaviour and sedentary behaviour may induce DNA methylation changes whereas DNA methylation in childhood may predispose for later traffic-related risky behaviour. For substance use associations in both temporal directions were observed. Risky sexual behaviour may result in hypomethylation in the *CALML*3 gene, which has been linked to certain behavioural/neurological phenotypes through its involvement in *MYO10* translation. Sedentary behaviour is associated with hypermethylation of CpG sites in the *NOTCH1*, *VAMP8* and *PLCD4* genes, all linked to developmental processes. *PYGO2*, which in these analyses is suggestive of a predisposition for subsequent risky behaviour in traffic, can influence the Wnt signalling pathway and therefore embryonic development.

There is very little data on associations between DNA methylation and risk behaviours, and as such the associations between DNA methylation at age 17 and MRB in mid-adolescence observed in our study cannot yet be compared to other studies. A previous study, conducted in the same population, assessed DNA methylation in relation to substance abuse, and concluded that birth was a potentially sensitive window of biological vulnerability (Cecil et al., 2016). Although we similarly found some evidence that DNA methylation may predispose for substance use, we observed hypermethylation of cg08761410 at birth in relation to substance use at age 17, which was a different CpG site to those observed in Cecil et al. (2016). In addition, we also observed associations between MRB at age 17 and DNA methylation at age 17, but not age 7 or at birth, indicating effects in adolescence and the possibility that behaviour may affect DNA methylation as well. Other possible explanations for the observed differences are that different subsets of the ARIES cohort were used in both studies, depending on the completeness of information for the analyses. Additionally, whereas the previous study used confirmatory factor analysis to extract three first-order factors of tobacco, cannabis and alcohol use, as well as a second-order factor for substance use, in our study instead we added up dichotomized (yes/no) risk behaviours because our main focus was on the total MRB score.

The causes underlying the development of MRB remain uncertain, but social processes, personality traits, and other individual factors, including biological processes, may be involved. It has been suggested that MRBs in adolescents may be the result of deficits in impulse-control, which has a neurological basis (Casey et al., 2008), and in agreement with that here we do observe some evidence of DNA methylation predisposing for traffic-related risk behaviour and substance use.

Similarly, DNA methylation has been associated with other lifestyle and environmental exposures as well, and although we adjusted our statistical models for what we believe to be the most important confounding factors, we cannot exclude residual confounding from other, non-included factors.

This study has several limitations. This study had reduced statistical power due to the relatively limited sample size of the study, which was further diminished as a result of missing values in the variables used to construct the risk scores as well as in the covariates, while also the study, uniquely, made use of (self-reported) behaviours rather than metrics with less measurement error. However, dichotomisation of risk behaviours was based on the literature. Although there are approximately 900 DNA methylation profiles at each of the three time points, for some association tests, the number with complete information is as low as 227. Moreover, missingness of information on risk behaviour is socially patterned and not “completely at random” (Relton et al., 2015). Missingness certainly reduces power, however, in other analyses of risk behaviour that have imputed for missing observations the direction of associations have not changed and often strengthened (for example: Gage et al., 2015; Mahedy et al., 2017). Nonetheless, even with the diminished sample size of this study we were still able to detect associations. Although multiple imputation could have been applied to increase sample size, current approaches are not feasible for genomic datasets including hundreds of thousands of measured variables, in our case variables corresponding to DNA methylation levels at over 480,000 CpG sites. In future, when feasible approaches have been developed, we plan to revisit these analyses.

A further limitation of the current analysis is the lack of directly measured blood cell type proportions. We therefore adjusted for cell count heterogeneity using estimates obtained using the method of Houseman et al. (2012). The method relies on having a reference dataset consisting of DNA methylation profiles of purified cell types. For peripheral blood a reference based on adult peripheral blood is available (Reinius et al., 2012), but not for childhood peripheral blood. However, for cord blood analysis, we were able to use a reference derived from cord blood (Gervin et al., 2016). Additional DNA methylation variation due to cell count heterogeneity would most likely have been represented in the surrogate variables also included as model covariates (McGregor et al., 2016).

Tissue specificity may also limit inferences from this study because levels of methylation vary between tissue types and may relate differently to traits and exposures. In the current study we have blood sample methylation but it may be have been more informative had we been able to test associations in a more relevant cell type such as neurons in the brain.

DNA methylation is only one epigenetic mechanism among a number of other interacting mechanisms including histone modifications and non-coding RNAs. It is possible that one or more of these other mechanisms is more strongly associated with risk behaviour. However, DNA methylation is a reasonable mechanism to consider in this initial study because it is the most stable and most readily available quantifiable epigenetic mark and has thus become the most widely studied (Bintu et al., 2016).

And finally, MRB was classified by aggregating self-reported individual risk behaviours identified by yes/no only, which will have impacted on measurement error. Previous work on substance use for example (Cecil et al., 2016), used confirmatory factor analysis to identify first and second order factors to define exposure, but this was for one risk behaviour only.

The main strength of this study is the unique resource which allowed for the assessment of genome-wide methylation profiles at different time points from birth to late adolescence linked to a detail phenotypic characterization, including information on a distinct set of risk behaviours representing the same time period, which enables assessment of the temporality of the associations. In these analyses we used three cross-sectional models to compare methylation patterns at birth, age 7 and in adolescence, but with better characterization of the dynamic elements of the human methylome (Ziller et al., 2013), longitudinal analyses will help to better elucidate persistent and reversible effects of (environmental) exposures as well as critical periods of effect (Mishra et al., 2009).

A further strength of this study is that, given the evidence that behaviours are known to cluster in individuals (MacArthur et al., 2012), in this study we could assess the cumulative effect of antisocial behaviour and delinquency, traffic-related risk behaviour, risky sexual behaviour, sedentary behaviour and substance use (MRB) together, in addition to the individual behaviours, on DNA methylation.

To our knowledge this is the first study to assess associations between DNA methylation and multiple risk behaviours in adolescents. This exploratory study provides the first evidence that MRB may result in changes to DNA methylation, but also provides evidence that DNA methylation may predispose for certain risk behaviours. As such, the results of this study indicate this topic requires further study, including a larger sample size – for example by combining different cohorts with comparable information, improved statistical methodology to take into account the longitudinal nature of the data as well as to address issues of missing data in a satisfactory manner, and which in turn will also allow for improved characterization of the temporal patterns of MRB development.
